# Full-scale cyclic testing of realistic reinforced-concrete beam-column joints

**DOI:** 10.1016/j.mex.2021.101409

**Published:** 2021-06-06

**Authors:** José Melo, Daniel A. Pohoryles, Tiziana Rossetto, Humberto Varum

**Affiliations:** a CONSTRUCT-LESE, Faculty of Engineering, Dept. of Civil Engineering, Univ. of Porto, Porto 4200-465, Portugal; formerly, Research Associate, Department of Civil, Environmental and Geomatic Engineering, EPICentre, Univ. College London, Chadwick Bldg., Gower St., London WC1E 6BT, UK; b European Commission, Joint Research Centre, Ispra 21027, Italy; formerly, Research Associate, Department of Civil, Environmental and Geomatic Engineering, EPICentre, Univ. College London, Chadwick Bldg., Gower St., London WC1E 6BT, UK; c Dept. of Civil, Environmental and Geomatic Engineering, EPICentre, Univ. College London, Chadwick Bldg., Gower St., London WC1E 6BT, UK; d CONSTRUCT-LESE, Faculty of Engineering, Department of Civil Engineering, Univ. of Porto, Porto 4200-465, Portugal

**Keywords:** Earthquake engineering, Cyclic testing, Reinforced concrete structures, Beam-column joint

## Abstract

The seismic performance of reinforced concrete (RC) structures are highly influence by the cyclic performance of the beam-column joints. The experimental seismic assessment of RC beam-column joints has been made essentially by cyclic tests performed on set-ups that do not totally simulate the real seismic loading and constrains conditions. A complex monitoring scheme is used to record the applied loads, reactions, joint distortion, strains on the reinforcement, lateral and axial displacements on the entire specimen, rotations and surface strains by using digital image correlation (DIC). The use of DIC is particularly important to record the strains on CFRP used to wrap the columns and beams. Based on the data recorded during the tests, it is possible compute moments; rotations and curvatures of the columns and beams; joint shear; dissipated energy by beams, columns and joint; yield displacement; ductility; peak-to-peak stiffness degradation; post-peak softening; and inter-cycle strength degradation. The innovative experimental set-up herein presented has the following advantages compared with others:

• Lateral loading applied on the top of the superior column and not on the beams
• Real scale specimens and the possibility of have transversal beams and slab
• Dead loads on the beams and columns with two different axial loads

## Graphical abstract

**Figure:**
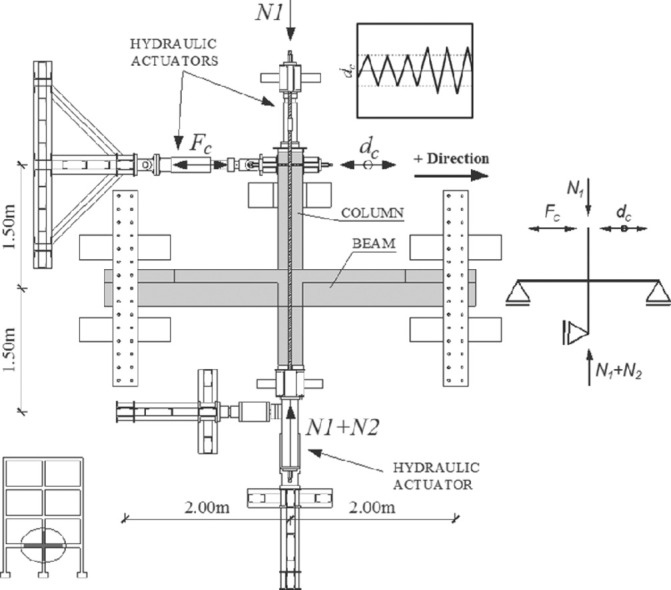

Specifications table

**Table:** 

Subject Area:	*•Engineering*
More specific subject area:	*Earthquake Engineering*
Method name:	*Full-scale cyclic testing of realistic reinforced-concrete beam-column joints.*
Name and reference of original method	*Melo J. Characterisation of the cyclic response of reinforced concrete elements with plain bars. PhD. Universidade de Aveiro, 2014.*
Resource availability	*The data system acquisition, servo-actuator displacement control and the acquisition software “Dynatester” were developed by IDMEC, INEGI at Faculty of Engineering, of Porto University. The servo-actuators were also created by IDMEC. The steel frame reactions were designed by the authors.*

## Method details

*The method described here-in is a methodology for full-scale cyclic testing of reinforced concrete (RC) beam-column joints with slab and transverse beams in the structural engineering laboratory environment of the University of Aveiro, Portugal. The method is an evolution from the previous testing set-ups for beam-column joints without slab at the same laboratory* [1,2]*.*

## Experimental set-up

The quasi-static cyclic test set-up for full-scale RC beam-column joints is presented in this section. The tests were carried out in the Laboratory of Aveiro University (Portugal) from November 2014 to October 2015 [3,4]. To the best knowledge of the authors, this corresponds to the first full-scale cyclic tests of realistic interior beam-column joints with slab and transverse beams, strengthened with FRP.

**Fig. 1:**
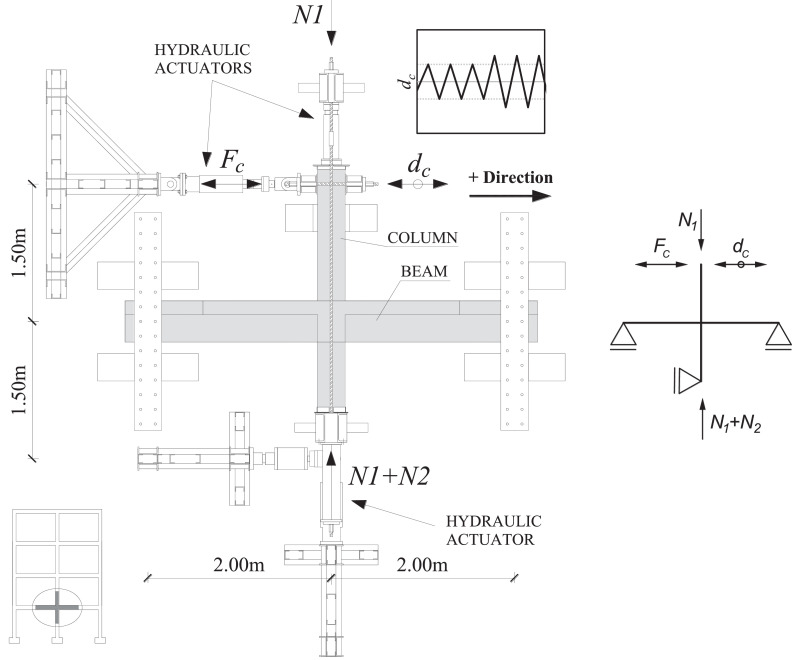
Test-set up with prototype structure and sample of loading protocol.

**Fig. 2:**
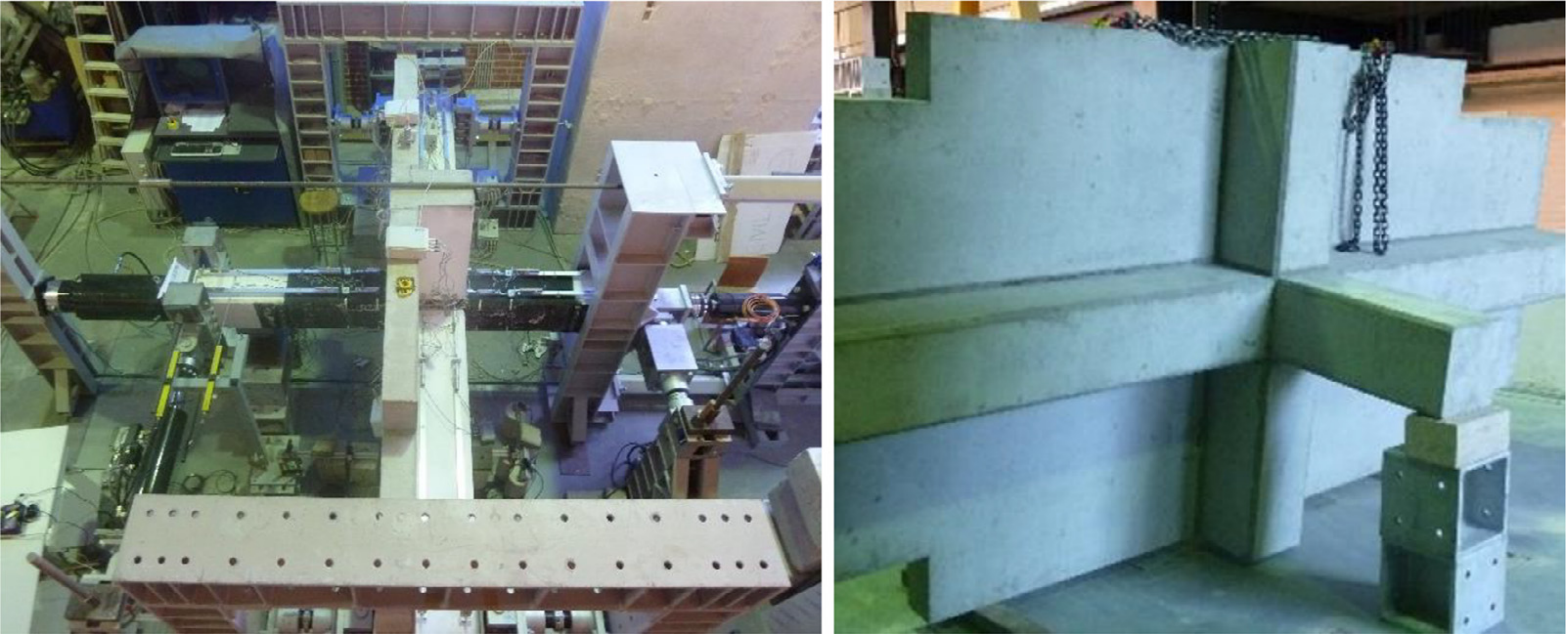
Pictures of the test set-up and specimen in the laboratory in Aveiro.

The loading set-up of the tests is shown in Fig. 1 and pictures of the set-up and specimen in the laboratory are shown in Fig. 2. A constant main axial load (*N1*) of 425 kN is applied through external pre-stress rods, which are pin-jointed at the hydraulic actuator at the top of the superior column and the bottom support of the inferior column. This axial load is applied before the beam supports are fastened. The value of *N1* is calculated for a second storey column in a typical residential four-storey RC frame in Europe.

**Fig. 3:**
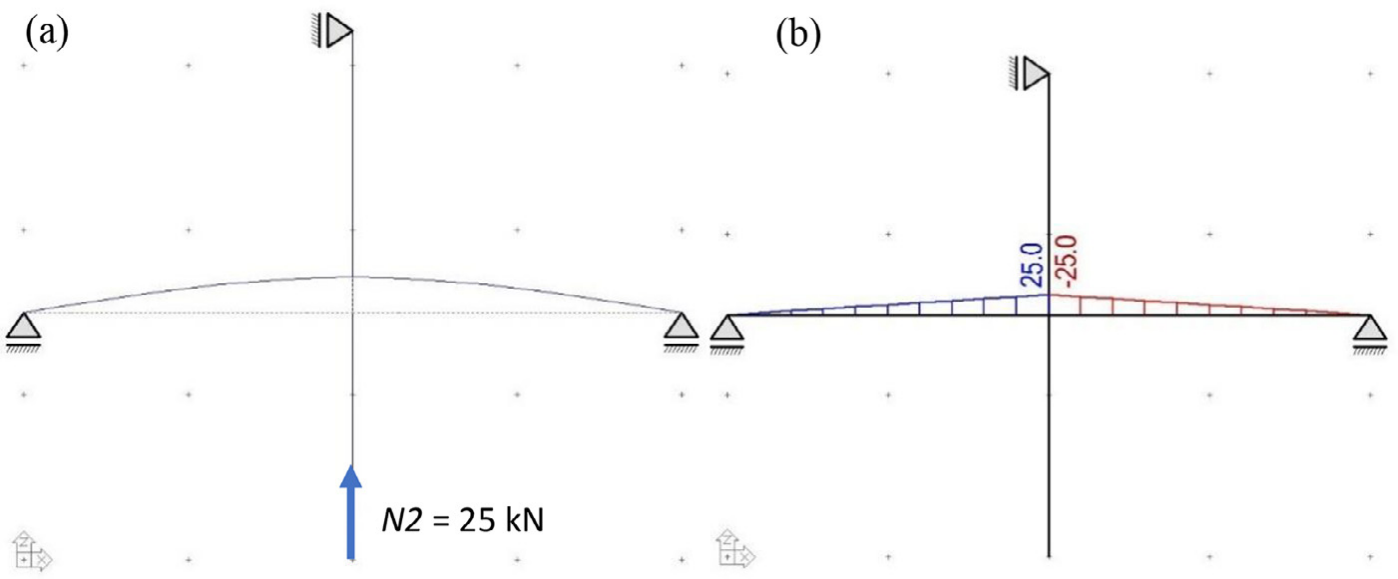
(a) Deflected shape and (b) moment diagram due to the second axial load (*N2*).

To induce a higher axial load in the first storey column, an additional axial load *(N2*) of 25 kN is applied at the inferior column. The second axial load is applied after beam supports are fastened so as to induce reaction forces in the beam supports, simulating moments from gravity loading, as shown in Fig. 3.

**Fig. 4:**
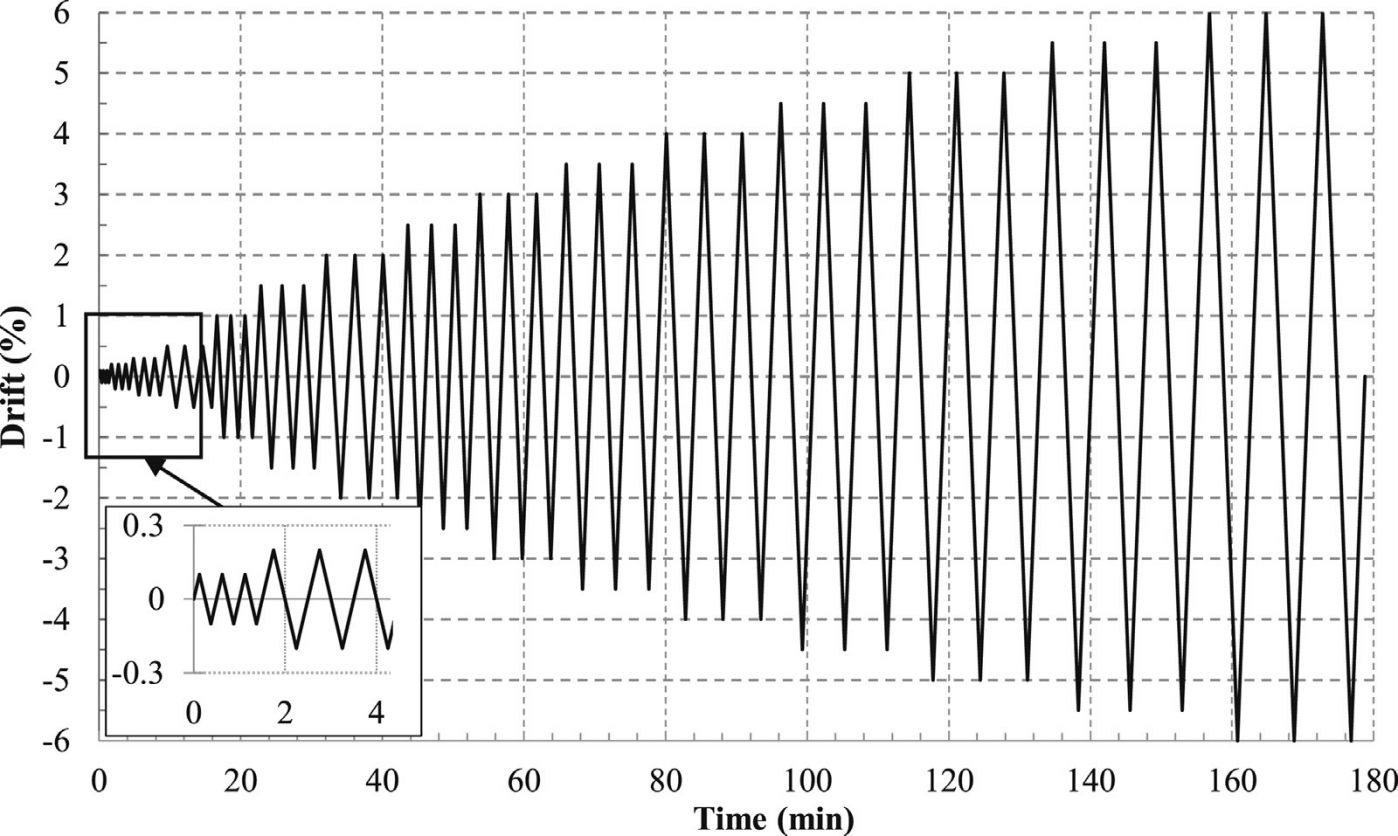
Applied displacement protocol for all experiments

Using a servo-hydraulic actuator, the lateral cyclic displacement (*d_c_*) or drift (Δ) protocol shown in Fig. 4 with three cycles per increment is applied at the top of the superior column, 1.5 m from the centre of the joint core. The drift values (in ± %) at each increment are: 0.1, 0.2, 0.3, then 0.5 up to 6.0 with 0.5 increments. The maximum lateral displacement at 6.0% drift is 180 mm. The rate of displacement application ranges from 0.1 mm/s in the first cycles up to 1.5 mm/s in the last cycles.

Due to the use of pre-stressing rods for the axial load application of *N1*, an eccentricity of the applied axial load occurs at high drift levels. The lateral load effectively applied, *F_l_*, needs to be calculated considering P-Δ effects from *N1*. This is done using the method described in the manual for the PEER structural performance database [5]:

(1) $$F_{l}=\;F_{c}+\;N1\cdot \frac{\delta_{ecc}}{L_{N}}$$

Here, *F_c_* is the applied lateral load from the horizontal hydraulic actuator, *δ_ecc_* is the measured eccentricity of the rod at the base of the superior column of the specimen, and *L_N_* the length from the base of the superior column to the top of the rod.

## Monitoring

**Fig. 5:**
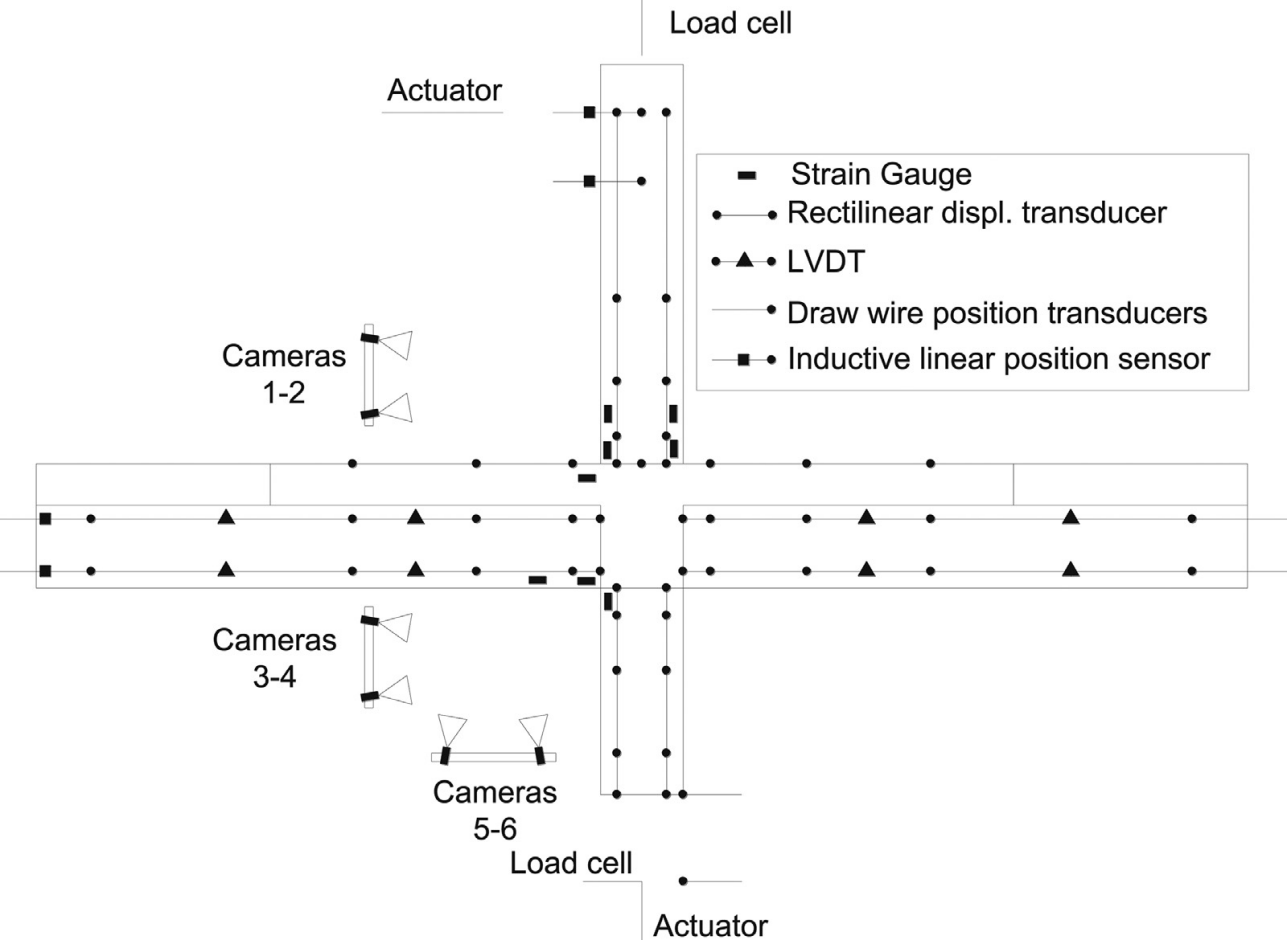
General arrangement of monitoring equipment for the beam-column joint tests.

The general arrangement of the monitoring equipment is shown in Fig. 5. The experiments are monitored using eight strain gauges (±0.6% accuracy) on the reinforcement (four on the superior column, one on the inferior column, two on the bottom beam bars and one on the top beam bars). In addition, one strain gauge on the top left FRP strand, 16 LVDT's (error < 0.025 mm), 28 rectilinear displacement transducers (error < 0.05 mm), four draw-wire position transducers (error < 0.5 mm), four inductive linear position sensors (error < 0.4 mm) and three pairs of cameras for stereoscopic 3D-digital-image correlation (DIC) are used to provide data on the deformation and damage evolution in the sub-assemblage. The DIC analysis is performed using the DaVis 8.2.3 software (LaVision).

**Fig. 6:**
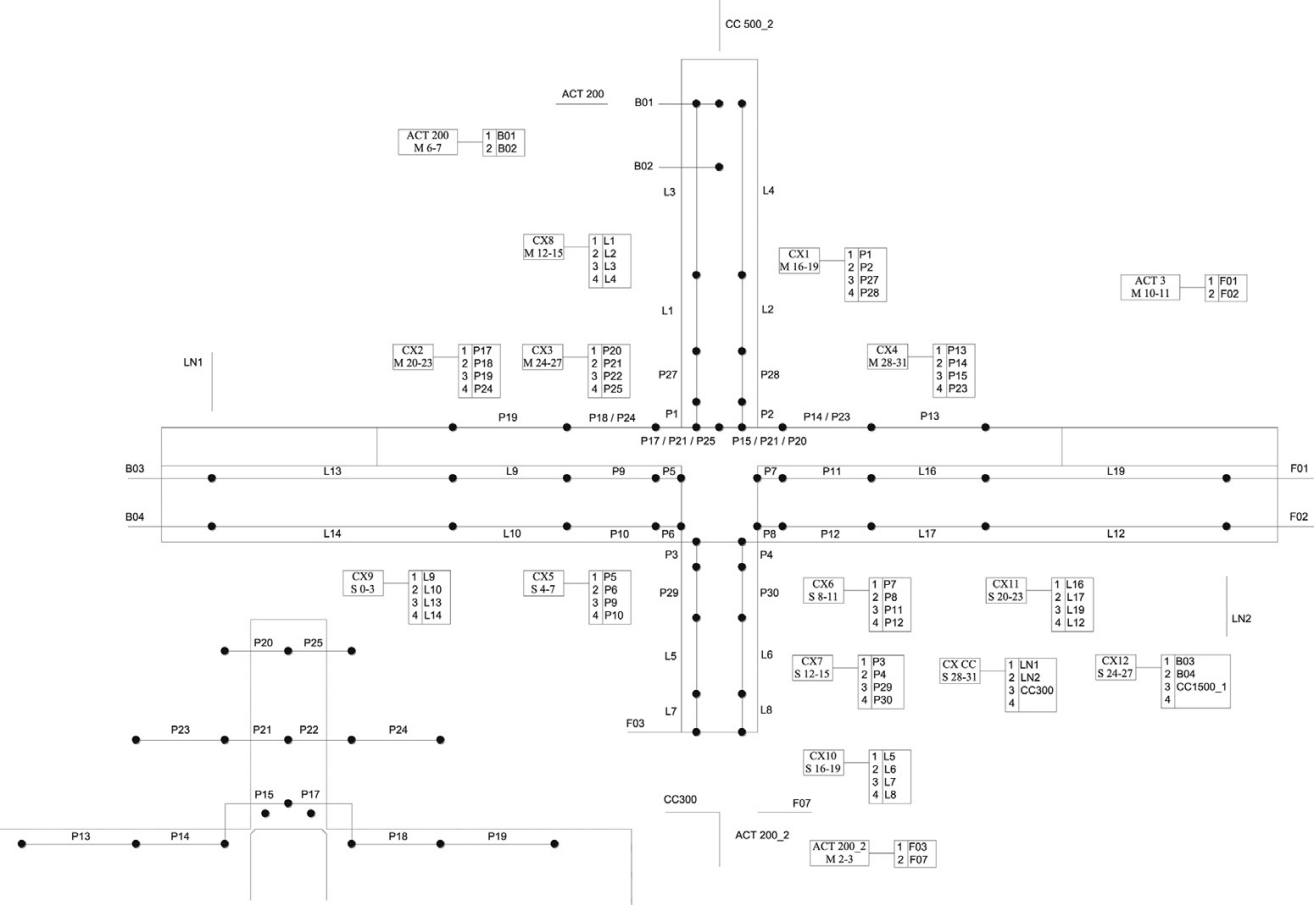
Monitoring set-up for specimens with slab.

**Fig. 7:**
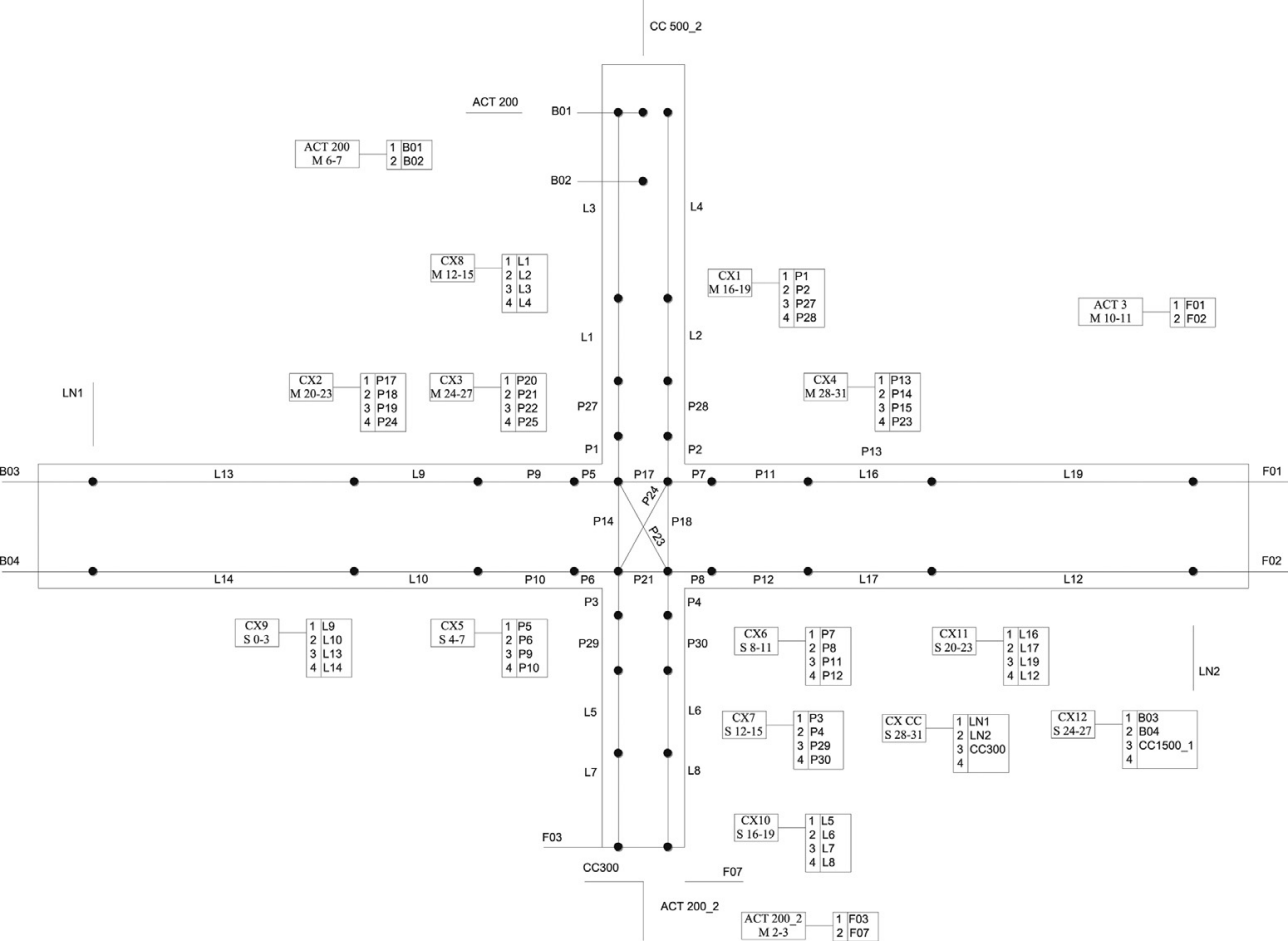
Monitoring set-up for specimens without slab.

Two different monitoring set-ups are used for the experiments due to changes in geometry and reinforcement detailing amongst the specimens tested. Fig. 6 shows the set-up used for specimens with slab and transverse beams. Fig. 7 shows the set-up used for specimens without slab.

## Diagnostics

A number of quantities are measured by the sensors presented within the experimental set-up. These include the value of lateral force (*F_c_*, in kN), lateral displacement (δ, m), strain gauge measurements on rebars (ε_s_) and the FRP strands (ε_FRP,strand_). However, other parameters need to be derived from these measurements in order to compare the response of different specimens or of different components within one specimen, and to compare this study with the literature. Hence, this section presents the diagnostics used to analyse and compare results from the experiments.

### Lateral storey drift

The value of drift is evaluated from the ratio of lateral displacement measured by sensor B01 (in m) and the sub-assembly storey height (3.0 m). Drift is presented as a percentage.

### Envelope of force-displacement curves

The envelope curves are created from the lateral force-displacement plots by joining the points at the end of the 1^st^ cycle of each drift level. The applied lateral force, *F_c_*, is measured in kN by the load cell in actuator ACT 200 at the top of the superior column. The maximum force is defined as the maximum value of *F_c_* measured during the test.

### Moments

#### Moment in columns

**Fig. 8:**
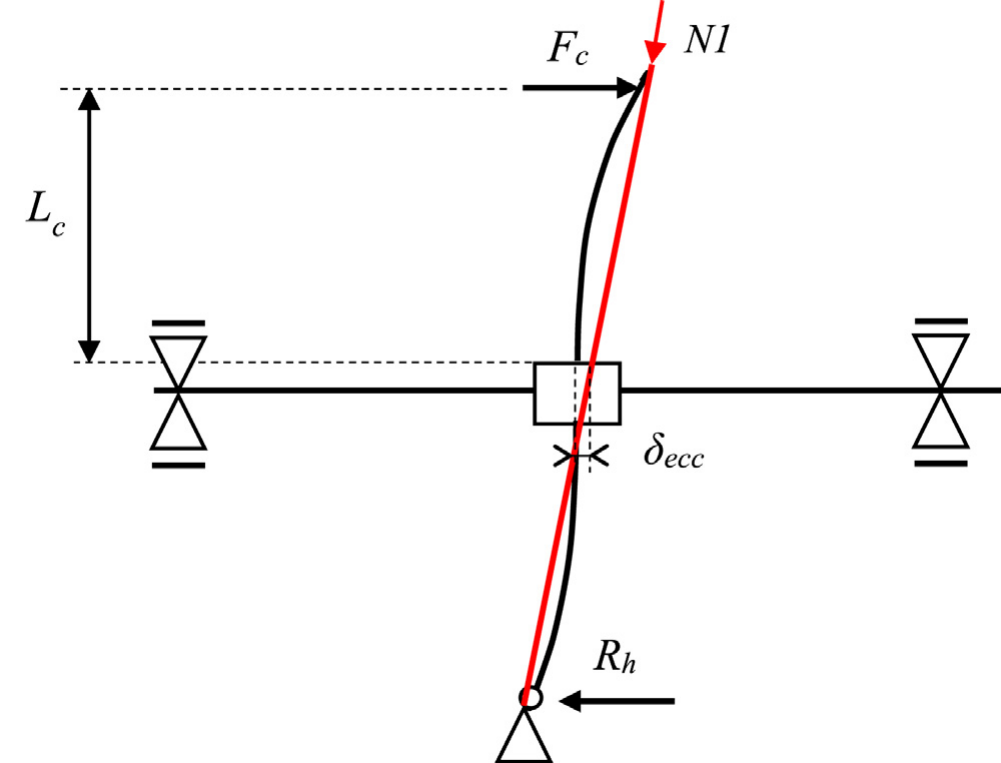
Schematic representation of the eccentricity in axial load application, *δ_ecc_*, with pre-stressing rods (indicated in red).

As shown in Fig. 8, due to the nature of the axial load application of *N1* with pre-stressing rods (represented by the red line in Fig. 8), an eccentricity of the applied axial load occurs at high drift levels.

The moment in the superior column, *M_c,sup_*, is hence a combination of the moment *M_l_* due to the lateral load, *F_c_*, applied at the top of the column, and the moment *M_ecc_* due to the eccentricity of axial load *N1*. The latter can be a significant contribution for large drift cycles and would also be present in a real structure undergoing large deformations. In the set-up, eccentricity of axial load was measured using string potentiometer F04 at the centre of the core joint, to measure the eccentricity of the steel rods (*δ_ecc_*) used to apply the axial load. The moment due to eccentricity is evaluated using the method described in the manual for the PEER structural performance database [5], given by equation (1):

(1) $$M_{ecc}=N_{1}\cdot \delta_{ecc}$$

The total superior column moment is hence:

(2) $$M_{c,sup}=M_{l}+\;M_{ecc}=\;F_{c}\cdot L_{c}+N_{1}\cdot \delta_{ecc}$$

Where *F_c_* is the applied lateral load from the horizontal hydraulic actuator ACT 200, *δ_ecc_* is the measured eccentricity of the rod at the base of the superior column of the specimen, and *L_c_* is the clear column height. *N1* is the applied axial load measured by the load cell in actuator CC 500_2.

For the inferior column, the moment is also evaluated as a combination of moment from the lateral force and the moment due to eccentricity, *M_ecc_*, but the lateral force is found from the resistance, *R_h_*, measured using the load cell CC300 at the base of the inferior column.

(3) $$M_{c,inf}=M_{l,i}+\;M_{ecc}=\;R_{h}\cdot L_{c}+N_{1}\cdot \delta_{ecc}$$

#### Moment in beams

**Fig. 9:**
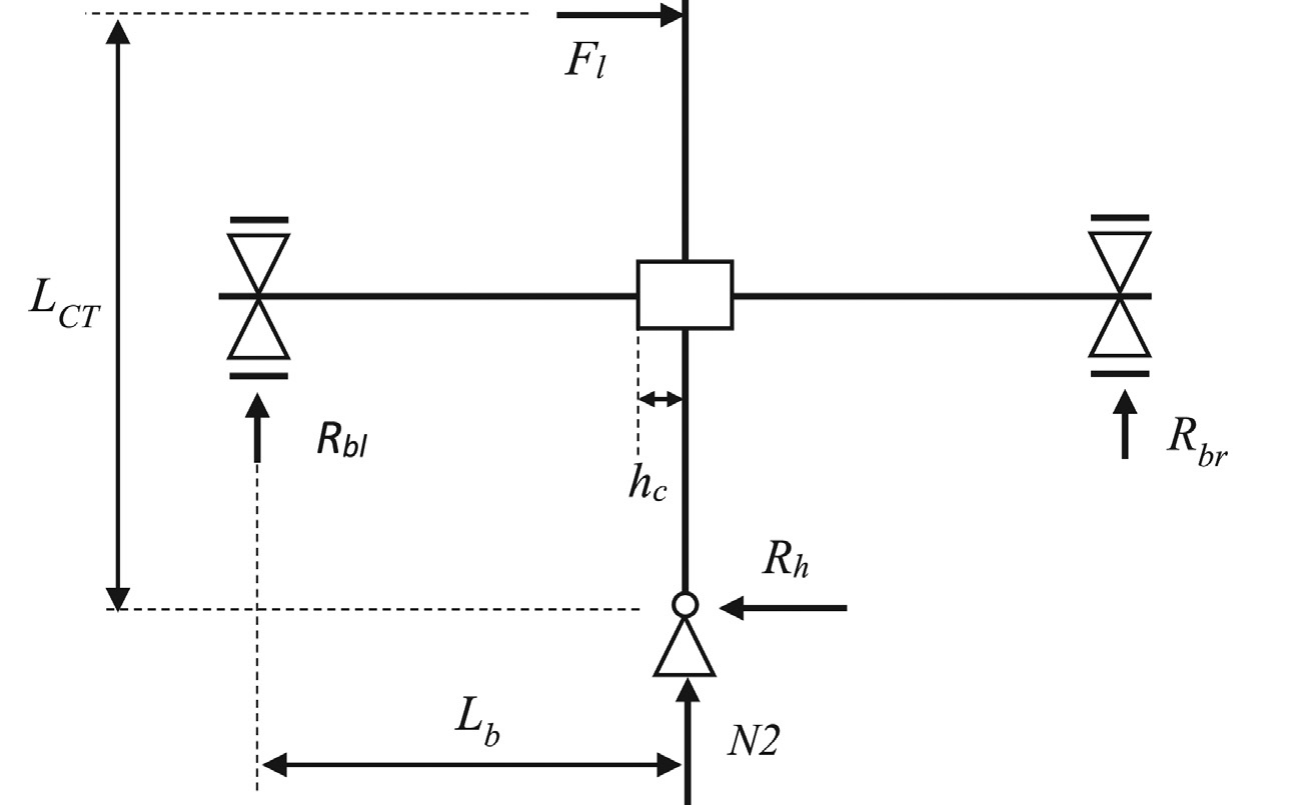
Loading and reactions for experimental set-up.

The beam moments are evaluated as a function of the superior column moment and the moment due to the second axial load, *N2*, of 25 kN. At the joint, the sum of column moments and beam moments has to equate. The loading and reactions in the specimens are shown in Fig. 9.

For vertical equilibrium:

(4) $$R_{bl}+R_{br}=-N2\;∴R_{bl}=-N2-R_{br}$$

Where *R_bl_* and *R_br_* are the reaction forces at the left and right beam support, respectively, as shown in Fig. 9. From equilibrium of moments, one can take the moments around the inferior column support:

$$F_{l}\cdot L_{CT}+R_{bl}\cdot L_{b}-R_{br}\cdot L_{b}=0$$

(5) $$∴R_{bl}-R_{br}=-\;\frac{F_{l}\cdot L_{CT}}{L_{b}}$$

Where *F_l_* is the total lateral load applied at the superior column, including the effect of eccentricity, *L_b_* the length between the beam supports and the centre of the joint (2.0 m), and *L_CT_* the length between the lateral load application and the inferior column support (3.0 m).

Hence, substituting for *R_bl_* from Eq. (4) in Eq. (5) and introducing the values of *N2, L_b_* and *L_CT_*, which are the same throughout all experiments:

$$∴-N2-R_{br}-R_{br}=-\;\frac{F_{l}\cdot L_{CT}}{L_{b}}$$

$$∴2\cdot R_{br}=\;\frac{F_{l}\cdot L_{CT}}{L_{b}}-N2$$

(6) $$∴R_{br}=\;\frac{F_{l}\cdot L_{CT}}{2\cdot L_{b}}-\frac{N2}{2}=\frac{F_{l}\cdot 3}{4}-\frac{25}{2}=\frac{3}{4}F_{l}-12.5$$

And:

(7) $$R_{bl}=-N2-R_{br}=-25-\left(\frac{3}{4}F_{l}-12.5\right)=-\frac{3}{4}F_{l}-12.5$$

Then the moments at the beam/joint interface are simply found from the following equations, where *h_c_*, the column cross-section height, is 0.3 m for all test specimens:

(8) $$M_{bl}=\;R_{bl}\cdot \left(L_{b}-\;h_{c}/2\right)=\;\left(-\frac{3}{4}F_{l}-12.5\right)\cdot 1.85$$

(9) $$M_{br}=\;R_{br}\cdot \left(L_{b}-\;h_{c}/2\right)=\;\left(\frac{3}{4}F_{l}-12.5\right)\cdot 1.85$$

### Joint shear (specimens without slab)

The joint shear force is determined based on equilibrium conditions and can be found from Equation (10). Joint shear is caused by the tension forces in the beam bars framing into the interior joint and the shear force from the column, *V_col_*, in the opposite direction (i.e. the applied lateral load, *F_c_*). The tension in the beam bars is found as the ratio of *M_b1_* and *M_b2_*, the moments in the beams on the left and right of the joint, with the lever arm, *j_d_*, defined as 0.75 *h_b_* [6]:

(10) $$V_{jh}=\frac{M_{b1}}{jd}+\frac{M_{b2}}{jd}-V_{col}$$

The shear stress in the joint core is commonly expressed as nominal shear stress or as principal tensile stresses. The horizontal shear stress (*ν_jh_*) in the joint can be calculated by equation (11), where *V_jh_* is the horizontal shear force in the joint, calculated by equation (12); *b_c_* is the width of the column; and *h_c_* is the depth of the columns.

(11) $$v_{jh}=\frac{V_{jh}}{b_{c}\cdot h_{c}}$$

Based on Mohr's circle, the principal tensile stresses (*p_t_*) at the mid-depth of the joint core is found from equation (12). Here *f_a_* is the nominal axial compressive stress on the column (equation (13). Note that compressive stresses are taken as negative.

(12) $$p_{t}=\frac{f_{a}}{2}+\sqrt{{\left(\frac{f_{a}}{2}\right)}^{2}+v_{jh}^{2}}$$

(13) $$f_{a}=\frac{N}{b_{c}\cdot h_{c}}$$

**Fig. 10:**
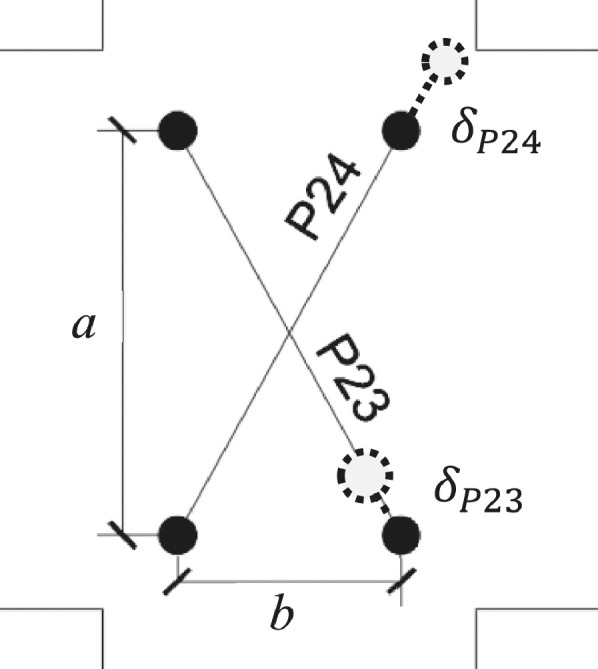
Transducers P23 and P24 used for the joint distortion calculation (dashed line indicates extension of transducer).

The joint distortion in radians, γ, is determined from the displacement readings δ_P23_ and δ_P24_ in the diagonal transducers *P23* and *P24* of the joint, as shown in Fig. 10.

The equation of joint distortion is given as [7]:

(14) $$\gamma =\frac{\sqrt{a^{2}+b^{2}}}{2ab}\left(\delta_{P23}+\delta_{P24}\right)$$

### Rotation and curvature

**Fig. 11:**
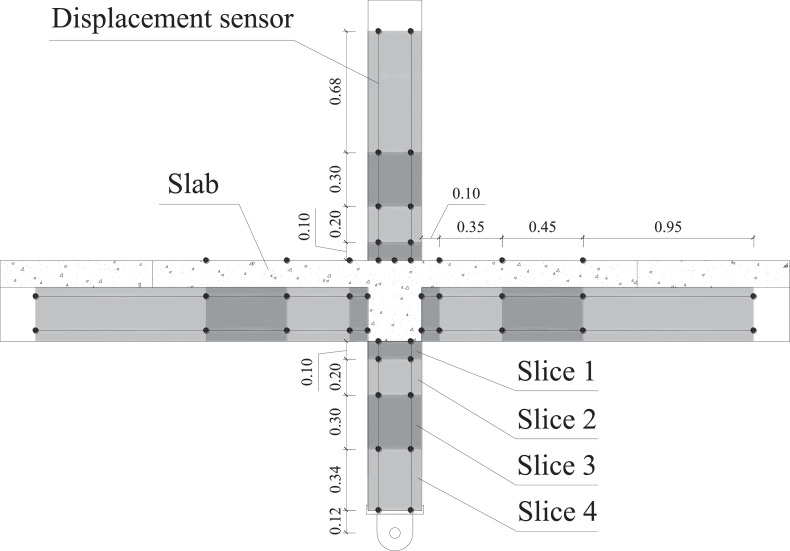
Details of 'slices' used for analysis of rotations and curvatures.

To assess the evolution of curvatures and rotation along the length of the columns and beams, each column and beam are divided into four lengths along the surface plane of the members, so-called ‘slices’, as shown in Fig. 11.

**Fig. 12:**
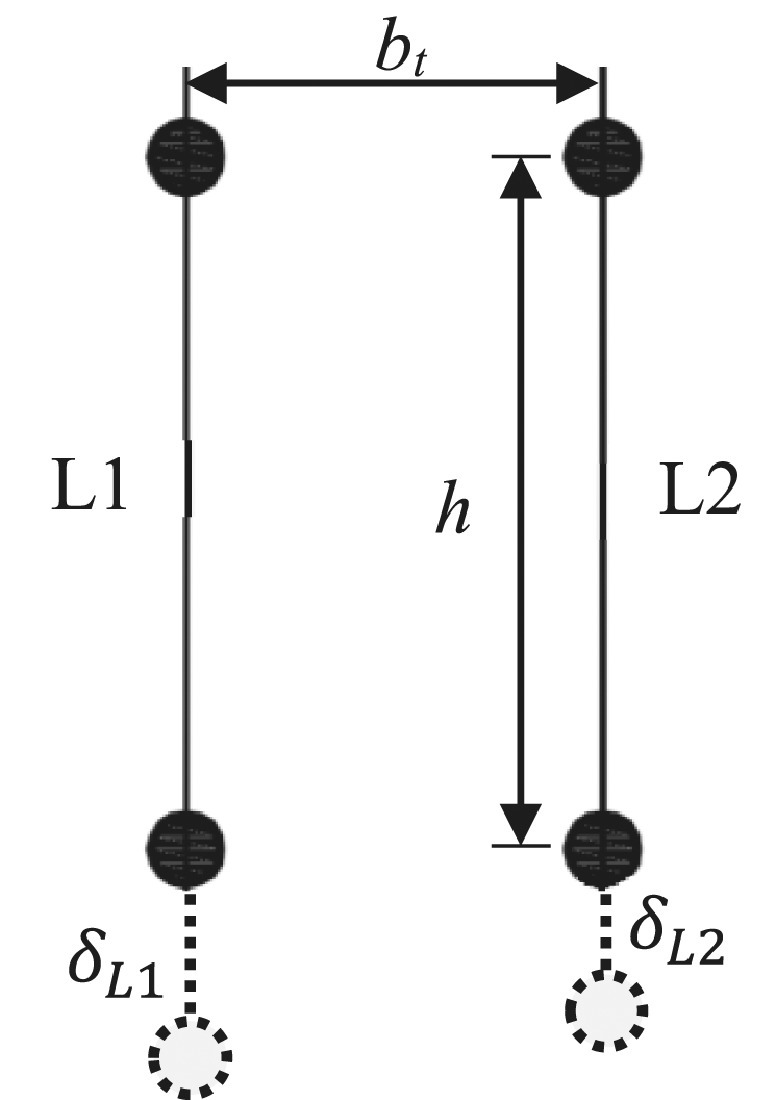
Transducers L1 and L2 used for the rotation and curvature calculation for slice 3, superior column (dashed line indicates extension of transducer).

The rotation, θ, in each slice is evaluated by comparing the deformations, *δ_i_*, of the two displacement transducers within the slice and dividing by the perpendicular distance between the two transducers, *b_t_*. For example, for slice 3 of the superior column, transducers *L1* and *L2* are used as shown in Fig. 12.

For the same example, the rotation, θ, is then given by:

(15) $$\theta =\frac{\delta_{L2}-\delta_{L1}}{b_{t}}$$

The curvature is then found by dividing the rotation by the slice length, *h*:

(16) $$\varphi =\frac{\delta_{L2}-\delta_{L1}}{b_{t}\cdot h}$$

The curvature in each slice is an average curvature over the length of the transducers. The pairs of transducers, with reference to the monitoring schemes shown in Monitoring section to calculate the rotation and curvature for each slice are summarised in Table 1.

**Table 1:** Summary of transducers used to evaluate rotations and curvatures in individual slices

Element	Slice	Slice length, *h*	Transducer pair	Distance between transducers, *b_t_*	
		[mm]		With slab	Without slab
Left beam	1	100	P5 – P6	180	320
	2	350	P9 – P10	180	320
	3	450	L9 – L10	180	320
	3a and 3b for C-EC8 only	225 each	L9 – L10 and P21 – P22	180	320
	4	950	L13 – L14	180	320
Right Beam	1	100	P7 – P8	180	320
	2	350	P11 – P12	180	320
	3	450	L16 – L17	180	320
	4	950	L19 – L12	180	320
Superior Column	1	100	P1 – P2	180	180
	2	200	P27 – P28	180	180
	3	300	L1 – L2	180	180
	4	675	L3 – L4	180	180
Inferior Column	1	100	P3 – P4	180	180
	2	200	P29 – P30	180	180
	3	300	L5 – L6	180	180
	4	340	L7 – L8	180	180

### Energy dissipation

**Fig. 13:**
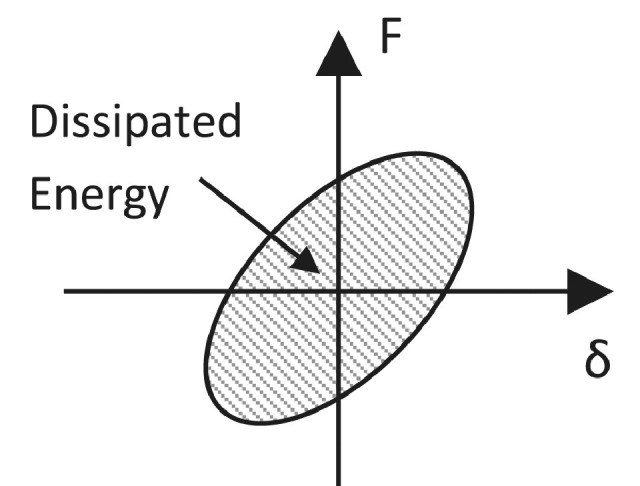
Schematic representation of global dissipated energy from force-displacement curve.

The global hysteretic energy dissipation of the specimens in units of kNm is defined as the area under the lateral force-displacement curves (see Fig. 13).

The cumulative dissipated energy, *E_diss_*, is hence defined as the integral of the force displacement plot. Using the trapezoidal rule, this can be calculated from:

(17) $$E_{diss}=\int_{0}^{\delta max}F\left(\delta \right).d\delta ≅\sum_{0}^{\delta_{i+1}=\;\delta max}\frac{\left(F_{i+1}+F_{i}\right)}{2}\times \left(\delta_{i+1}-\delta_{i}\right)$$

Where *δ_i_* and *F_i_* are the measured lateral displacement and applied force, respectively, at each level of displacement, *i*.

Energy dissipation is an indication of increased damage, as inelastic deformations lead to large energy dissipation. Higher dissipated energy developing over large drift levels is an indication of an improved, ductile, seismic behaviour [8]. High energy dissipation at low levels of drifts, combined with a low value of ultimate drift is however an indication of damage at very early loading stages of the experiments. This in turn translates into a poorer seismic behaviour with significant damage occurring for smaller earthquakes.

The contribution of the individual members (beams, columns and joint) to the global energy dissipation, *E_diss,member_*, is calculated from the moment-rotation curves at different sections along the length of the members. Each column and beam are divided into four slices, as shown previously. The moment and rotation at the centre of each slice is calculated assuming constant moment and rotation within each slice. As most of the inelastic deformations occur in the first two slices, that are smaller in length, this approximation is adequate. This method is common in the literature and good agreement is found in previous studies [1,2].

(18) $$E_{diss,member}=\sum_{slice\;=\;1}^{4}\int_{0}^{\theta max}M\left(\theta \right).d\theta ≅\sum_{slice\;=\;1}^{4}\sum_{0}^{\theta_{i+1}=\;\theta max}\frac{\left(M_{i+1}+M_{i}\right)}{2}\times \left(\theta_{i+1}-\theta_{i}\right)$$

Where θ*_i_* and *M_i_* are the previously defined rotations and moments in the slice, respectively, at each level of displacement, *i*.

For specimens with slab, due to the experimental set-up containing transverse beams, the joint is not instrumented and the energy dissipated by joint deformations is hence approximated as the remainder between the difference of the global dissipated energy and the energy dissipated by columns and beams. For the specimens without slab and transverse beam, this approximation is verified by also calculating the energy dissipated from joint distortion (see Section Energy dissipated by joint distorcion).

To understand the evolution of the contribution of the individual members to the global energy dissipation, plots showing the proportion of dissipated energy for beams, columns and joint at the end of the last cycle for every level of drift are produced. Following rules of capacity design, a better seismic behaviour is indicated by a higher proportion of beam participation.

### Energy dissipated by joint distortion (specimens without slab)

For the two specimens without slab, C-noSLT and C-noSLT-RT-B, the energy dissipated in the joint is evaluated from the joint shear force and joint shear distortion calculated in Section Joint shear. The energy dissipated by the joint. *E_diss,j_* is then found from the area under the joint shear – joint distortion curve, i.e. the integral of this plot, which is approximated using the trapezoidal rule:

(19) $$E_{diss,j}=\int_{0}^{\gamma max}V_{jh}\left(\gamma \right).d\gamma ≅\sum_{0}^{\gamma_{i+1}=\;\gamma max}\frac{\left({V_{jh}}_{i+1}+{V_{jh}}_{i}\right)}{2}\times \left(\gamma_{i+1}-\gamma_{i}\right)$$

Where γ*_i_* and *V_jh_*_,_*_i_* are the previously defined joint distortion and horizontal joint shear force, respectively, at each level of displacement, *i*.

### Ductility factor

The ability of a structure to dissipate energy by undergoing large plastic deformations is characterised by its ductility. In this study the ultimate displacement ductility, μ_Δu_, is chosen to characterise the global ductility:

(20) $$\mu_{\Delta u}=\frac{\delta_{u}}{\delta_{y}}=\frac{\Delta_{u}}{\Delta_{y}}$$

**Fig. 14:**
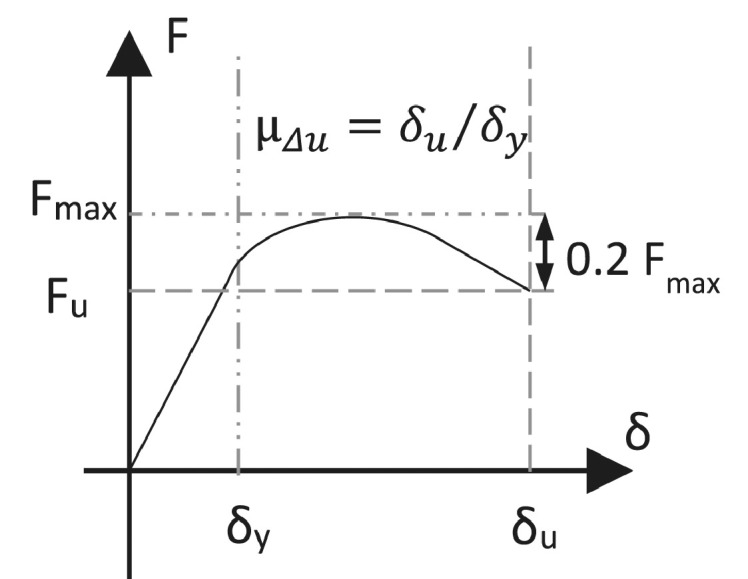
Schematic representation of displacement ductility from the force-displacement envelope curve.

Where δ_u_ and Δ_u_ are the ultimate displacement and drift of the specimen, respectively, and δ_y_ and Δ_y_ the yield displacement and drift of the specimen, respectively. This is represented graphically in Fig. 14, with the yield and ultimate displacement points defined as in Sections Yield displacement and drift and Ultimate displacement and drift. The ultimate displacement corresponds to displacement where a 20% strength reduction relatively to the maximum strength occurs, as adopted by Park et al. [9].

### Yield displacement and drift

The ultimate displacement ductility is dependent on the value of the Δ_y_, the yield drift. As indicated in Monitoring section, the strain gauges are placed near the interfaces of the members to the joint, hence at the locations of maximum moment and where yield of rebars is occurs first. In this study, yield drift for each element is hence defined as the first drift at which the measured strain at one of the strain gauge locations exceeds the yield strain ε_y_ of the longitudinal steel reinforcing bars (taken as 0.21% for the 12 mm rebars and 0.3% for the high strength 16 and 25 mm rebars used for C-EC8). To reduce the potential for physical errors due to inadequate strain gauge application, multiple strain gauges are applied in each member, as outlined in Monitoring section.

Using strain gauge readings is a commonly used experimental method for defining yield when adequate monitoring is used [e.g.: 10,11]. While alternative methods for determining the yield displacement based on the force-drift envelope curves exist (e.g. using an equivalent elasto-plastic system with equal energy dissipation), the importance relies in using a consistent means of defining yield, and hence ductility, for all specimens.

Following the rules of capacity design, it is desirable for yield of beam bars to occur before that of column bars. A higher value of yield drift for column bars is hence seen as an improvement when assessing the repair and retrofit interventions.

### Ultimate displacement and drift

The ultimate drift, Δ_u_, is defined according to Park et al. [9] as the level of global drift after the maximum force (*F_max_*) is reached in the specimen, at which the lateral force capacity of the specimens drops by 20%. Note that the ultimate point is extrapolated from the force-drift envelope to ensure consistency between the experiments. This is a commonly accepted definition of reaching the ultimate state [12].

### Peak-to-peak stiffness

**Fig. 15:**
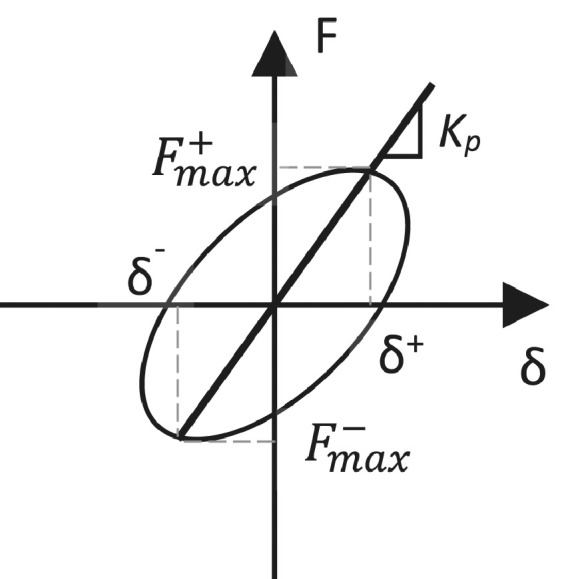
Schematic representation of the peak-to-peak stiffness calculation.

As shown in Fig. 15, the peak-to-peak lateral stiffness, *K_p_*, expressed in units of kN/mm, is defined as the slope of the line between the maximum positive and negative force ($F_{max,i}^{+}$ and $F_{max,i}^{-}$) at the first cycle of each level of displacement, *i*:

(21) $$K_{p,i}=\;\frac{\left|F_{max,i}^{+}\right|+\left|F_{max,i}^{-}\right|}{\left|\delta_{i}^{+}\right|+\left|\delta_{i}^{-}\right|}$$

Plots of lateral stiffness against drift are used to highlight stiffness degradation with increased drift for the specimens. This diagnostic is used extensively in the literature [e.g.: [13], [14], [15], [16], [17]] and is an important parameter in evaluating the effectiveness of a retrofit. A lower rate of degradation in stiffness corresponds to a better seismic behaviour of the sub-assemblies, as loss of stiffness is not desirable. For pre-damaged and repaired specimens, a lower initial stiffness is expected and this is visualised using plots of peak-to-peak stiffness against drift.

### Post-peak softening

**Fig. 16:**
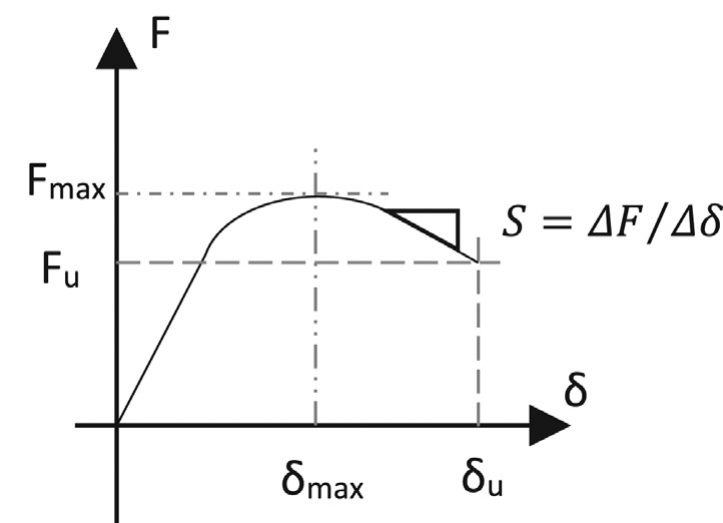
Schematic representation of the post-peak softening calculation from the force-displacement envelope.

The post-peak softening, *S*, is a characteristic of the behaviour of a structure after the maximum load, and is shown diagrammatically in Fig. 16. The value of *S*, determined in units of kN/mm, is found from the slope between the maximum force, *F_max_*, and the ultimate force, *F_u_*, at their respective levels of lateral displacement. A higher softening is hence associated with a steeper strength reduction from *F_max_* to *F_u_*, and hence a lower residual strength for the structure at any level of drift.

### Inter-cycle strength degradation

**Fig. 17:**
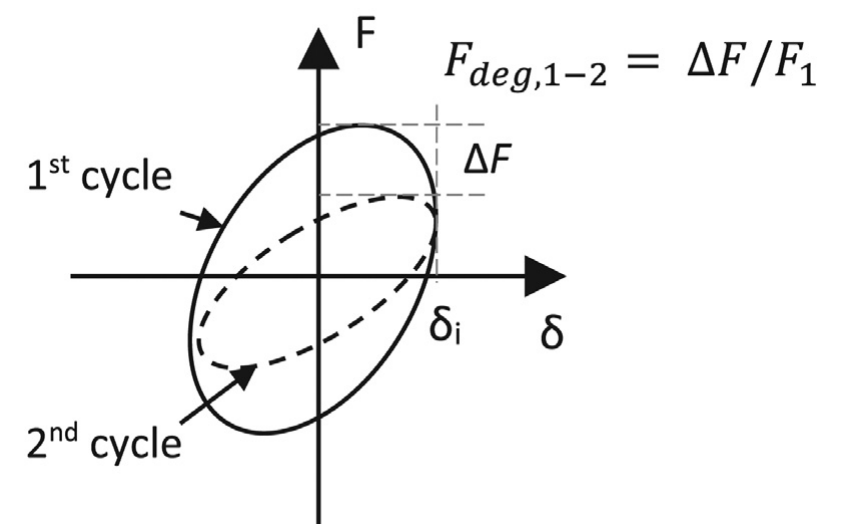
Schematic representation of the inter-cycle strength degradation calculation.

The strength degradation between cycles 1 and 2 (*F_deg_*_,1-2_) and 1 and 3 (*F_deg,_*_1-3_) is evaluated at each level of drift. This corresponds to the reduction in lateral load capacity at the end of each cycle (Fig. 17). It is an important parameter to understand the seismic behaviour of the specimens, as in real earthquakes structures undergo repeated cycles of load. A low reduction in strength upon repeated loading is hence desirable. Strength degradation is determined as a percentage of reduction from the 1^st^ cycle and is plotted against increased drift levels. An average is taken between the values for loading in positive and negative directions. Strong reductions in strength between cycles are usually associated to brittle damage in the specimens, such as joint damage [2].

## Method validation

**Fig. 18:**
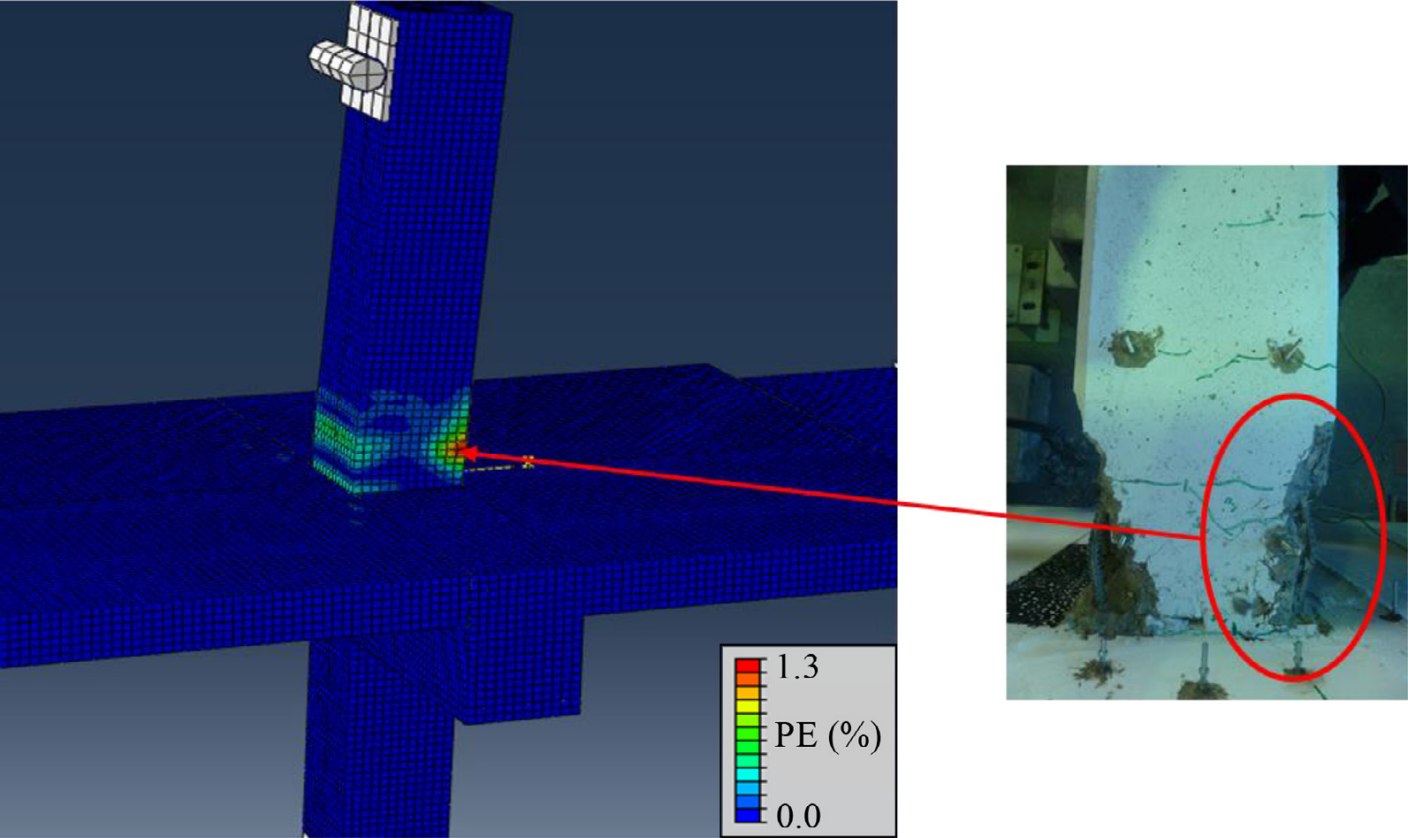
Expected damage in specimen from FE-modelling and observed experimental damage for specimen with slab.

**Fig. 19:**
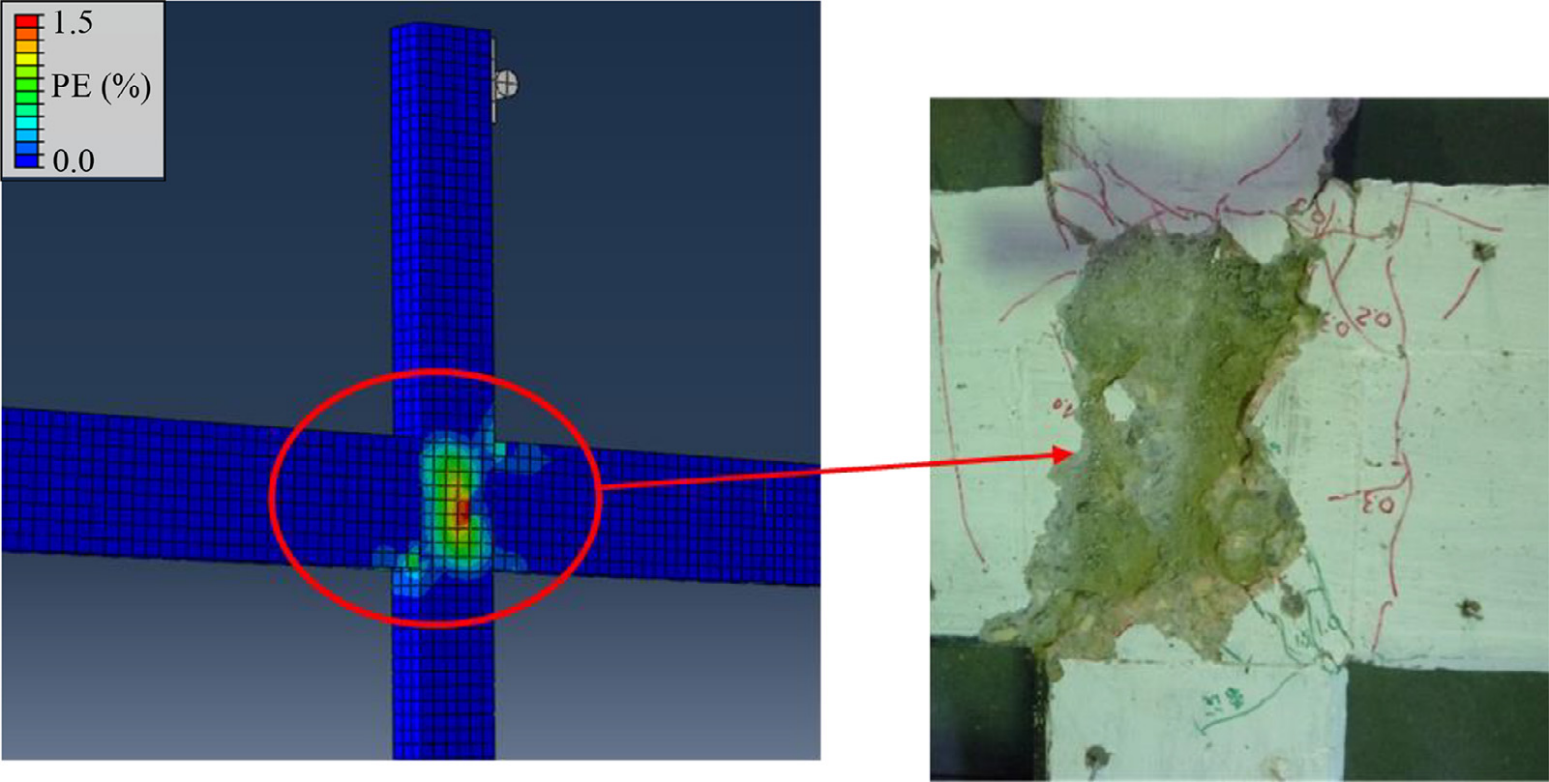
Expected damage in specimen from FE-modelling and observed experimental damage for cruciform specimen.

In order to validate the methodology, the expected damage and failure mechanisms from a detailed finite-element study [18] were used to assess the experimentally obtained damage in the RC beam-column joints. Detailed results of the experiments are published elsewhere [3,19], however the validation of the method can be seen when comparing the damage obtained in Fig. 18 and Fig. 19, where damage is indicated by in terms of plastic strain (PE) in the concrete damaged plasticity model [20].

As can be observed, the experimental framework reproduces expected behavior very well. It is further worth noting that yielding of beam bars occurred first in the top of the beams, due to larger hogging than sagging moments near the joint. This result confirms the importance of reproducing realistic load conditions experimentally, inducing an additional moment through the second axial load, N2, hence simulating the effect of gravity loading on the beams.

## Declaration of Competing Interest

The authors declare that they have no known competing financial interests or personal relationships that could have appeared to influence the work reported in this paper.
